# Associations between physical physique/fitness in children and bone development during puberty: a 4-year longitudinal study

**DOI:** 10.1038/s41598-022-17623-z

**Published:** 2022-08-04

**Authors:** Arata Akaike, Daisuke Suzuki, Shinya Okuyama, Yutaro Kudo, Hiroyasu Shimizu, Sara Takanashi, Kohei Makino, Junichi Yokoyama, Shigeyuki Nakaji

**Affiliations:** 1 Department of Social Medicine, Hirosaki University Graduate School of Medicine, 5 Zaifu-cho, Hirosaki, Aomori 036-8562 Japan; 2 Faculty of Sport Science, Nippon Sport Science University, Tokyo, 158-8508 Japan

**Keywords:** Health care, Quality of life, Epidemiology

## Abstract

Bone growth is most remarkable during puberty. This study aimed to clarify the effects of physique and physical strength on bone mineral density and bone metabolism markers during puberty to help improve bone growth during puberty and prevent future osteoporosis. There were 277 pubertal participants (125 boys and 152 girls) in this survey from 2009 to 2015, all aged 10/11 and 14/15 years. The measures included physical fitness/physique indices (such as muscle ratio etc.), grip strength, bone density (osteo sono-assessment index, OSI), and bone metabolism markers (bone-type alkaline phosphatase and type I collagen cross-linked N-telopeptide). At 10/11-years-old for girls, a positive correlation was found between body size/grip strength and OSI. At 14/15-year-old for boys, all body size factors/grip strength were positively correlated with OSI. The change in body muscle ratio was positively correlated with change in OSI for both sexes. The height, body muscle ratio and grip strength at 10/11-year-old were significantly associated with OSI (positively) and bone metabolism markers (negatively) at 14/15-year-old for both sexes. Adequate physique building after 10/11 years for boys and before 10/11 years for girls may be effective in increasing peak bone mass.

## Introduction

A healthy life expectancy is the average length of time a person can live in good health with independence in activities of daily living, as proposed by the World Health Organization (WHO) in 2000^1^. In Japan, the gap between healthy life expectancy and average life expectancy is now more than 10 years^2^. Therefore, the "National Health Promotion Movement in the 21st Century (Healthy Japan 21)" was established to help extend healthy life expectancy^3,4^. To achieve this aim, it is necessary to delay the time when people will nursing care. The primary causes of care needs in Japan are locomotive syndrome, frailty, and osteoporosis^5^. Moreover, control of metabolic syndrome, childhood obesity, frailty, and locomotive syndrome are measures to prevent the need for nursing care^6^.

It is well-known that habitual moderate exercise is necessary for good health. In order to exercise, the locomotor system, comprising bones, joints, and muscles, must be healthy. Therefore, in 2007, the Japanese Orthopedic Association defined "locomotive syndrome" as "a condition in which mobility is impaired due to musculoskeletal disorders and [wherein] the risk of requiring long-term care in the future is high,"^7^ and has since been working on preventive measures. However, according to a 2021 white paper aging, fractures and diseases of the musculoskeletal system^8^ remain the most common cause of care needs in Japan, accounting for a quarter of all care needs.

In particular, osteoporosis, which causes bone fractures, reportedly affects 7.9% of men and 22.9% of women over the age of 40 years in Japan^9,10^. Early detection and treatment seem to be the most important ways to prevent osteoporosis. Bbone mineral density (BMD) assessment is essential for early detection and treatment. Dual energy X-ray absorption (DXA) has traditionally been used as an indicator for bone evaluation among various radiological methods. However, since fractures reportedly occur even with high BMD, the 2000 National Institutes of Health (NIH) Consensus Conference^11^ recommended adding bone quality as a measure of bone assessment. However, evaluating bone quality is still difficult.

One of the methods to evaluate BMD is ultrasonic measurement (quantitative ultrasound, QUS)^12–15^. Studies have also shown that QUS and DXA resuits correlate with each other^16–27^. However, QUS is noninvasive, non-radioactive, and can be used to evaluate pregnant women and children. Moreover,it has the distinct advantage over DXA that it is mobile.

Bone is resorbed by osteoclasts and formed by osteoblasts. BMD is maintained if the bone metabolic turnover is normal and the balance between bone resorption and bone formation is maintained.

In contrast, abnormalities in bone metabolic turnover induce decreased BMD. Therefore, to detect osteoporosis at an early stage, bone metabolism markers are used in Japan to evaluate bone metabolic turnover, bone metabolism markers are independent BMD-related indicators^11^ that, include bone formation and resorption markers. The Fracture Intervention Trial (FIT), which has fracture prevention as an endpoint, reported that BMD is a bone formation marker rather than a bone resorption marker^16,28^. In this study, bone metabolism markers were also measured to objectively examine the dynamics of bone metabolism. These included bone formation markers (bone-type alkaline phosphatase, BAP) and bone resorption markers (type I collagen cross-linked N-telopeptide, NTX).

Adolescence is the peak height velocity age (PHVA), when bones grow rapidly, and BMD reaches its maximum value (peak bone mass, PBM) at approximately 20 years of ago^16^.

One way to prevent osteoporosis is to have a high PBM. However, because the details of bone metabolism in adolescents are unknown, specific measures to increase PBM cannot be proposed.

Accordingly, this study aimed to clarify the effects of physique and physical strength on BMD and bone markers during puberty, when bone growth is most-active.

The features of this study are as follows:

1. This is a 4-year cohort study from the fifth elementary grade to the third-year of junior high school.
2. Bone metabolism markers were measured in third-year junior high school students.
3. Body composition was measured and examined separately using the body fat ratioand body muscle ratio.

## Methods

### Participants

Participants included teens boys and girls who took the Iwaki Health Promotion Project Elementary and Junior High School Health Survey in both fifth grade in elementary school and third year of junior high school.

Participants were selected as follows:

1. Four elementary and junior high schools in the Iwaki district of Hirosaki City, located in the north of Japan, were chosen. The survey was conducted in autumn.
2. From 2009 to 2011, surveys and measurements were conducted on fifth graders (10/11-year-old students) who provided their consent, as did of their parents. Of the 395 subjects, 361 participated in the survey, with a participation rate of 91.4%.
3. From 2013 to 2015, surveys and measurements were conducted on third-year junior high school students (14/15-year-old students) who provided their consent, as did their parents. Of the 415 subjects, 380 participated in the survey, with a participation rate of 84.3%.
4. A total of 323 students who fulfilled (2) and (3) were selected.
5. Of the 323 participants, those with a history of cardiovascular disease, diabetes, dyslipidemia, or hypertension, those on medication, those with a history of bone fractures, those with a history of calcaneal fractures, and those with missing values in the analysis items were excluded. A total of 277 pubescent teens (125 boys and 152 girls) were included in the analysis.

The components of the survey were a questionnaire, bone density measurement, blood test (bone metabolism marker), and physical fitness measurement. The survey was conducted within one day in elementary school and 1–2 days in junior high school. The survey took a total of 5 days.

### Measures

#### Questionnaire

A self-administered questionnaire was provided in advance. Participants were asked to complete it out with their parents or guardians, and the questionnaire was collected on the day of the measurement. Four public health experts reviewed the responses, and if they had any questions, they checked with the child or his/her parents. The questionnaire items included age, gender, medical history, current medical history, and medication status.

#### Anthropometric evaluation (height and body composition measurements)

Height and body composition measurements were taken on the day of the survey as part of the physique assessment.

The body composition measurements included body weight, body fat ratio (% fat), and body muscle ratio (% muscle). A body composition analyzer based on the bioelectrical impedance method (TBF-110; Tanita Corporation, Tokyo) was used for measurement. This device uses multiple frequencies of 5 kHz, 50 kHz, 250 kHz, and 500 kHz and has been used in many studies on adults^29–31^. The instrument was designed to measure participants who are at least 110 cm tall and at least 6 years of age.

#### Bone evaluation^32,33^

BMD is a major component of bone toughness. BMD evaluation was performed by QUS using an ultrasonic bone evaluation device (AOS-100NW; Aloka Co., Ltd., Tokyo, Japan). The measurement site was the calcaneus bone, which was evaluated through the acoustic bone assessment value (Osteo Sono-Assessment Index, OSI). The speed of sound (SOS) and transmission index (TI) were measured by this device and then used to calculate OSI. SOS was used to measure calcification and bone density^34,35^, whereas TI was used to measure broadband ultrasound attenuation, a bone quality assessment index^12,15^. OSI was calculated using the formula:

$${\text{OSI}} = {\text{TI}} \times {\text{SOS}}^{{2}} ,$$

thus reflecting the characteristics of both SOS and TI. Hence, OSI is considered one of the global index values in acoustic bone assessment^36^.

#### Muscle strength evaluation

To evaluate muscle strength, we used grip strength, which is considered^37,38^ to reflect whole-body muscle strength. We followed the method of the "New Physical Fitness Test"^39^ by the Sports Agency of the Ministry of Education, Culture, Sports, Science and Technology.

The Smedley type grip strength meter(T. K. K. 5401; Takei Scientific Instruments Co., Ltd., Niigata, Japan). was used to measure grip strength, with grip width adjusted such that the proximal interphalangeal joint of the second finger was flexed at 90°. For measurement, the limb position was a standing position with both legs open, the pointer of the grip strength meter was held so that it faced outward, and the shoulders were slightly laterally displaced to not touch the body. Subsequently, the participant was asked to grasp the grip strength meter with full force while exhaling. During measurement, the participants were asked to hold the grip strength meter still while maintaining the basic posture. Measurement was performed twice on each hand and, were taken alternately on the left and right hands, with the best value being adopted.

#### Measurement of bone metabolism markers

Blood samples were taken from children in their third year of junior high school in the early morning on an empty stomach, and blood analysis was outsourced to LSI Medience Co., Ltd. The company also conducted the measurements for bone formation (BAP) and bone resorption markers (NTX) using the CLEIA (Chemiluminescent enzyme immunoassay) method.

### Statistical analyses

All analyses were performed according to sex.

The measures obtained from the fifth grade of elementary school and those from the third-year of junior high school were compared using paired t-tests.

To examine potential confounding factors, the correlations between OSI and height, % fat, % muscle, and grip strength were confirmed for each grade using a partial correlation coefficient. For participants in their third year of junior high school, the correlation between OSI, BAP, and NTX was confirmed using the partial correlation coefficient.

To investigate the effect of changes in physique and physical strength on OSI from the fifth grade of elementary school to the third year of junior high school, the changes in % fat, % muscle, and grip strength in relation to the change in OSI were examined using a multiple regression analysis. For this analysis, the change in OSI was used as an objective variable, and the change in each item was used as an explanatory variable.

Odds ratios with 95% confidence interval were calculated using logistic regression analysis to assess the relationship between physique and physical parameters during the fifth grade of elementary school and bone metabolism (OSI, BAP, and NTX) during the third year of junior high school.

Height, % fat, % muscle, and grip strength were used as physique/physical fitness indicators for students in fifth-grade elementary school, with each indicator used to divid students into tertiles of low-value, medium-value, and high-value groups.

SPSS 16.0J software (SPSS Inc., Chicago, IL, USA) was used for statistical analyses, and a p-value < 0.05 was considered statistically significant.

### Ethical considerations

This study was conducted according to the principles of the Declaration of Helsinki.

The purpose of the study, the right to withdraw from the study at any point, and the data management methods (including data privacy protection and data anonymity) were thoroughly explained to all participants, and written consents were obtained from either the participants themselves or from their parents/caregivers.

The Iwaki Health Promotion Project Elementary and Junior High School Health Survey was approved by the Institutional Review Board of the Graduate School of Medicine, Hirosaki University (approval numbers 2009-048, 2010-084, 2011-111, 2013-339, 2014-060, and 2015-075).

This study was registered in the University Hospital Medical Information Network (UMIN-CTR, https://www.umin.ac.jp; examination name: Iwaki Health Promotion Project Medical Examination; and UMIN Exam ID: UMIN000040459).

## Results

### Physical fitness and physique indices, bone mineral density, and bone metabolism markers (Tables 1 and 2)

**Table 1:** Baseline characteristics of participants (boys, N = 125).

Characteristics	10/11-year-olds	14/15-year-olds	p-value
Height (cm)	142.5 ± 6.7	167.8 ± 6.8	0.000
Body weight (kg)	37.0 ± 9.4	57.7 ± 10.9	0.000
Body fat ratio (%)	17.2 ± 10.8	17.9 ± 7.7	0.160
Body muscle ratio (%)	28.3 ± 3.6	44.2 ± 4.8	0.000
Grip strenght (kg)	17.8 ± 3.4	32.1 ± 5.4	0.000
OSI (× 10^5^)	2.6 ± 0.4	2.9 ± 0.4	0.000
BAP (µg/L)	–	57.6 ± 38.7	–
NTX (nmol BCE/mmol)	–	77.7 ± 31.5	–

Paired-samples t-test.

SD, standard deviation; p, probability value; N, number of participants; OSI, osteo-sono assessment index; BAP, bone specific alkaline phosphatase; NTX, type I collagen cross-linked N-telopeptide.

**Table 2:** Baseline characteristics of participants (girls, N = 152).

Characteristics	10/11-year-olds	14/15-year-olds	p-value
Height (cm)	144.5 ± 6.7	157.2 ± 5.7	0.000
Body weight (kg)	37.4 ± 7.4	51.7 ± 8.3	0.000
Body fat ratio (%)	20.0 ± 7.1	30.2 ± 6.7	0.000
Body muscle ratio (%)	27.9 ± 3.5	33.6 ± 3.2	0.000
Grip strength (kg)	17.6 ± 3.3	22.6 ± 3.9	0.000
OSI (× 10^5^)	2.5 ± 0.2	3.0 ± 0.4	0.000
BAP (µg/L)	–	23.0 ± 15.5	–
NTX (nmol BCE/mmol)	–	35.4 ± 13.8	–

Paired-samples t-test.

SD, standard deviation; p, probability value; N, number of participants; OSI, Osteo-sono assessment index; BAP, bone specific alkaline phosphatase; NTX, type I collagen cross-linked N-telopeptide.

Among boys, all items except % fat significantly increased, whereas among girls, all items significantly increased. Bone metabolism marker values in the third year of junior high school were also significantly higher in boys than in girls, indicating that bone metabolism during this period was more active in boys than in girls.

### Correlation in each grade (Tables 3 and 4)

**Table 3:** Correlation between each parameter (boys, N = 125).

		Height		% fat		% muscle		Grip strength		OSI		BAP		NTX	
		r	p	R	p	r	p	r	p	r	p	r	p	r	p
10/11-year-olds	Height			0.287	0.001	0.886	0.000	0.451	0.000	0.075	0.204				
	% fat	0.287	0.001			0.485	0.000	0.348	0.000	0.058	0.259				
	% muscle	0.886	0.000	0.485	0.000			0.594	0.000	0.080	0.186				
	Grip strength	0.451	0.000	0.348	0.000	0.594	0.000			0.060	0.254				
	OSI	0.075	0.204	0.058	0.259	0.080	0.186	0.060	0.254						
14/15-year-olds	Height			0.060	0.252	0.732	0.000	0.363	0.000	0.148	0.049	− 0.445	0.000	− 0.406	0.000
	% fat	0.060	0.252			0.460	0.000	0.256	0.002	0.274	0.001	− 0.025	0.390	− 0.209	0.010
	% muscle	0.732	0.000	0.460	0.000			0.651	0.000	0.429	0.000	− 0.122	0.088	− 0.339	0.000
	Grip strength	0.363	0.000	0.256	0.002	0.651	0.000			0.390	0.000	− 0.142	0.057	− 0.454	0.000
	OSI	0.148	0.049	0.274	0.001	0.429	0.000	0.390	0.000			− 0.328	0.000	− 0.331	0.000
	BAP	− 0.025	0.390	− 0.122	0.088	− 0.142	0.057	− 0.328	0.000	− 0.445	0.000			0.366	0.000
	NTX	− 0.209	0.010	− 0.339	0.000	− 0.454	0.000	− 0.331	0.000	− 0.406	0.000	0.366	0.000		

Partial correlation coefficient.

The 10/11-year-olds were fifth-grade elementary school students, whereas the 14/15-year-olds were third-year junior high school students.

N, Number of participants; r, partial correlation coefficient; p, probability value; % fat, body fat ratio; % muscle, body muscle ratio; OSI, osteo-sono assessment index; BAP, bone specific alkaline phosphatase; NTX, type I collagen cross-linked N-telopeptide.

**Table 4:** Correlation between each parameter (girls, N = 152).

		Height		% fat		% muscle		Grip strength		OSI		BAP		NTX	
		r	p	R	p	r	p	r	p	r	p	r	p	r	p
10/11-year-olds	Height			0.207	0.005	0.885	0.000	0.659	0.000	0.405	0.000				
	% fat	0.207	0.005			0.463	0.000	0.130	0.055	0.188	0.010				
	% muscle	0.885	0.000	0.463	0.000			0.672	0.000	0.525	0.000				
	Grip strength	0.659	0.000	0.130	0.055	0.672	0.000			0.356	0.000				
	OSI	0.405	0.000	0.188	0.010	0.525	0.000	0.356	0.000						
14/15-year-olds	Height			− 0.095	0.242	0.781	0.000	0.532	0.000	0.154	0.058	− 0.075	0.359	0.093	0.255
	% fat	− 0.095	0.242			0.210	0.009	0.071	0.385	0.157	0.054	− 0.178	0.028	− 0.202	0.013
	% muscle	0.781	0.000	0.210	0.009			0.619	0.000	0.374	0.000	− 0.097	0.236	− 0.094	0.250
	Grip strength	0.532	0.000	0.071	0.385	0.619	0.000			0.242	0.003	− 0.137	0.093	− 0.061	0.455
	OSI	0.154	0.058	0.157	0.054	0.374	0.000	0.242	0.003			− 0.067	0.411	− 0.189	0.020
	BAP	− 0.075	0.359	− 0.178	0.028	− 0.097	0.236	− 0.137	0.093	− 0.067	0.411			0.376	0.000
	NTX	0.093	0.255	− 0.202	0.013	− 0.094	0.250	− 0.061	0.455	− 0.189	0.020	0.376	0.000		

Partial correlation coefficient.

The 10/11-year-olds were fifth-grade elementary school students, whereas the 14/15-year-olds were third-year junior high school students.

N, Number of participants; r, partial correlation coefficient; p, probability value; % fat, body fat ratio; % muscle, body muscle ratio; OSI, Osteo-sono assessment index; BAP, bone specific alkaline phosphatase; NTX, type I collagen cross-linked N-telopeptide.

For girls in the fifth grade, a positive correlation was found between body size/grip strength and OSI. However, this trend was not observed among boys.

For third-year junior high school boys, all body size factors/grip strength were positively correlated with OSI and negatively correlated with NTX and /BAP. In contrast, this trend was less pronounced among girls.

### Relationship between changes in physical fitness and physique indices and changes in BMD over 4 years (Tables 5 and 6)

**Table 5:** Relationship between changes in physical fitness/physique indices and changes in BMD over 4 years (boys, N = 125).

Objective variable	Explanatory variable	B	Standard error	β	*p*	Adjusted R^2^
OSI	Change in height	− 0.065	0.013	− 0.618	0.000	0.354
	Change in % fat	0.003	0.008	0.036	0.750	
	Change in % muscle	0.058	0.018	0.408	0.001	
	Change in grip strength	0.009	0.009	0.086	0.321	

Multiple regression analysis.

Adjusted by values at 10/11-year-olds (fifth-grade elementary school students).

B, partial regression coefficient; β, standard partial regression coefficient; p, probability value; BMD, bone mineral density; N, Number of participants; OSI, osteo-sono assessment index; % fat, body fat ratio; % muscle, body muscle ratio.

**Table 6:** Relationship between changes in physical fitness/physique indices and changes in bone mineral density over 4 years (girls, N = 152).

Objective variable	Explanatory variable	B	Standard error	β	p	Adjusted R^2^
OSI	Change in height	− 0.033	0.013	− 0.458	0.013	0.172
	Change in % fat	0.002	0.007	0.022	0.804	
	Change in % muscle	0.069	0.023	0.510	0.003	
	Change in grip strength	0.011	0.0011	0.099	0.303	

Multiple regression analysis.

Adjusted by values at 10/11-year-olds (fifth-grade elementary school students).

B, partial regression coefficient; β, standard partial regression coefficient; p, probability value; BMD, bone mineral density; N, Number of participants; OSI, osteo-sono assessment index; % fat, body fat ratio; % muscle, body muscle ratio.

The change in body muscle ratio was positively correlated with the change in OSI among both sexes.

### Relationship between physical fitness and physique index at 10/11 years of age and bone metabolism at 14/15 years of age (Tables 7 and 8)

**Table 7:** Odds ratios of physical fitness/physique index at 10/11 years of age for OSI/ bone metabolism at 14/15 years of age (boys, N = 125).

		Tertile value (n)	OSI			BAP			NTX		
			OR	95% CI	p	OR	95% CI	p	OR	95% CI	p
10/11-year-olds	Height (cm)	126.7–139.5 (41)	–	–	–	–	–	–	–	–	–
		139.6–145.0 (42)	1.841	[0.500–6.771]	0.359	1.004	[0.993–1.015]	0.517	0.993	[0.979–1.007]	0.302
		145.1–158.5 (42)	7.057	[1.952–25.506]	0.003	0.989	[0.978–1.001]	0.078	0.963	[0.945–0.981]	0.000
	% fat (%)	3.0–10.3 (41)	–	–	–	–	–	–	–	–	–
		10.5–18.8 (41)	1.612	[0.485–5.356]	0.172	0.993	[0.982–1.004]	0.229	1.000	[0.987–1.014]	0.998
		19.0–57.0 (41)	3.387	[3.387–10.872]	0.040	0.984	[0.972–0.996]	0.008	0.965	[0.947–0.984]	0.000
	% muscle (%)	20.85–26.90 (32)	–	–	–	–	–	–	–	–	–
		26.95–29.45 (44)	6.095	[1.412–26.310]	0.015	0.997	[0.986–1.008]	0.586	0.984	[0.969–0.999]	0.033
		29.50–40.90 (49)	24.581	[5.350–112.929]	0.000	0.980	[0.967–0.992]	0.002	0.945	[0.924–0.967]	0.000
	Grip strength (kg)	9.4–15.9 (32)	–	–	–	–	–	–	–	–	–
		16.0–18.0 (44)	1.570	[0.356–6.923]	0.551	0.981	[0.967–0.995]	0.008	0.993	[0.979–1.007]	0.309
		19.0–28.0 (49)	10.764	[2.505–46.260]	0.001	0.969	[0.955–0.984]	0.000	0.977	[0.962–0.993]	0.004

Logistic regression analysis.

The 10/11-year-olds were fifth-grade elementary school students, whereas the 14/15-year-olds were third-year junior high school students.

OR, odds ratio; p, probability value; % fat, body fat ratio; % muscle, body muscle ratio; OSI, osteo-sono assessment index; BAP, bone specific alkaline phosphatase; NTX, type I collagen cross-linked N-telopeptide.

**Table 8:** Odds ratios of physical fitness / physique index at 10/11 years of age for OSI/ bone metabolism at 14/15 years of age (girls, N = 152).

		Tertile value (n)	OSI			BAP			NTX		
			OR	95% CI	p	OR	95% CI	p	OR	95% CI	p
10/11-year-olds	Height (cm)	127.9–141.9 (54)	–	–	–	–	–	–	–	–	–
		142.1–147.4 (49)	10.349	[2.761–38.792]	0.001	0.973	[0.947–1.000]	0.047	0.962	[0.933–0.992]	0.014
		147.5–163.3 (49)	9.789	[2.616–36.629]	0.001	0.944	[0.913–0.975]	0.001	0.945	[0.912–0.979]	0.002
	% fat (%)	8.6–16.5 (51)	–	–	–	–	–	–	–	–	–
		16.6–20.5 (50)	0.904	[0.312–2.623]	0.853	0.974	[0.949–1.001]	0.056	0.965	[0.936–0.995]	0.021
		20.6–47.5 (51)	1.099	[0.387–3.120]	0.859	0.953	[0.924–0.983]	0.002	0.961	[0.931–0.991]	0.013
	% muscle (%)	18.55–26.20 (50)	–	–	–	–	–	–	–	–	–
		26.30–29.35 (48)	8.010	[1.980–32.410]	0.004	0.956	[0.926–0.986]	0.004	0.944	[0.912–0.978]	0.001
		29.50–39.10 (54)	19.066	[4.666–77.911]	0.000	0.948	[0.918–0.979]	0.001	0.934	[0.901–0.969]	0.000
	Grip strength (kg)	9.8–15.8 (51)	–	–	–	–	–	–	–	–	–
		16.0–19.0 (53)	3.542	[1.079–11.628]	0.037	0.987	[0.963–1.011]	0.286	0.971	[0.943–0.999]	0.040
		19.3–25.0 (48)	5.541	[1.652–18.592]	0.006	0.952	[0.922–0.983]	0.003	0.931	[0.895–0.969]	0.000

Logistic regression analysis.

The 10/11-year-olds were fifth-grade elementary school students, whereas the 14/15-year-olds were third-year junior high school students.

OR, odds ratio; p, probability value; % fat, body fat ratio; % muscle, body muscle ratio; OSI, osteo-sono assessment index; BAP, bone specific alkaline phosphatase; NTX, type I collagen cross-linked N-telopeptide.

There was a tendency for significantly higher odds ratios in the maximum height, % fat, % muscle,and grip strength groups for higher OSI in third year of junior high school at third-year junior high school among fifth-grade students.

Furthermore, higher height, %fat, %muscle, and grip strength in fifth grade for both males and females tended to have significantly lower odds ratios for the BAP and NTX results in ninth grade.

## Discussion

Bone repeated formation and resorption occur throughout life. These bone metabolic activities are regulated by various hormones^40–46^ and cytokines. Bone growth has two peaks: the primary growth phase until the age of 5 years and the secondary growth phase during the teenage years. During the secondary growth phase, the bone finishes growing in the long-axis direction, the epiphyseal line closes, and the trabecular bone becomes denser, which improves BMD. The participants in this study were in the period of secondary sexual characteristics development, when the secretion of sex hormones is active and factors that affect bone metabolism are entangled. Rauchenzauner et al.^47^ reported that bone metabolism during puberty exhibits a large variation with according to age and sex, and that both BAP and tartrate-resistant phosphatase, a bone resorption marker, decrease after the age of 15 years. However, a study focusing on these factors in Japanese adolescents has not yet been conducted. Reports of trends in bone metabolism markers and factors associated with DXA among Japanese adolescents are also very limited^48^. One reason for this is the unwillingness of parents and guardians to allow invasive tests such as blood sampling and exposure to radiation on their children in conditions that do not involve diagnosis or treatment.

The main results of this study are:

1. For girls in fifth grade, a positive correlation was found between body size/grip strength and OSI. However, this trend was not observed among boys. This suggests that the development of body size during early adolescence influences OSI among girls.
2. For third year junior high school boys, all body size factors/grip strength were positively correlated with OSI. In contrast, this trend was less pronounced among girls, with only change in % muscle and grip strength positively correlating with OSI. Change in body muscle ratio was positively correlated with the change in OSI among both sexes. These results suggest that for boys, growth in body size/muscle power from the fifth grade of elementary school to the third year of junior high school influences OSI.
3. The height, body muscle ratio and grip strength in fifth-grade elementary school were significantly positively associated with OSI and significantly negatively associated with bone metabolism markers in the third year of junior high school for both sexes. These findings suggest that the development of body size (height and body muscle ratio) and grip strength in early adolescence influences OSI and bone metabolism.

The peak height velocity age (PHVA) during the second growth period among Japanese people is observed at 13 years old for boys and 11 years old for girls, with the rate of growth in height being higher in boys^49^. At age 17 for boys and 15 for girls, the closure of the epiphyseal line begins and BMD increases toward PBM. Considering the results of the present study in light of this background, we suggest that increasing height, muscle mass, and muscle strength by fifth grade for girls and after fifth grade for boys, is important for increasing PBM.

Previous studies in growing children and adolescents have reported that both bone resorption and bone formation markers eventually become elevated^50^. This may reflect active bone metabolism.

The relationship between bone metabolism and BMD has been the subject of many studies among adults^51,52^. Although there are some reports^53–56^ with slightly different trends in men, an overview of the results of previous studies can be summarized as follows: “Bone metabolism markers increase during growth and subsequently, decrease until the 40 s, remaining unchanged until old age”.

In Japan, the reference values for BAP are 3.7–20.9 µg/L for healthy men and 2.9–14.5 µg/L for healthy premenopausal women. The reference values for NTX are 9.5–17.7 nmol BCE/L for healthy men and 7.5–16.5 nmol BCE/L for healthy premenopausal women^57^. Compared to these reference values, both markers were elevated in our study among thirdyear junior high school students and this was more pronounced in boys. This indicates that bone metabolism is active in thirdyear students, especially in boys. The reason for the gender difference could be that the third-year boys are still in the growth phase and the epiphyseal line has not yet closed, whereas in girls, the epiphyseal line is nearer to closure during this period. That is, boys in the third year of junior high school are still growing and bone growth is active, whereas girls are at the end of their bone growth period and reaching bone maturity. The trends of bone metabolism markers obtained in this study are consistent with peak height velocity age in the Japanese population^16^.

Furthermore, the results of this study suggested that those with large and strong physique and physical strength in the fifth grade of elementary school had the peak of bone metabolism at a younger age.

However, one limitation of the present study is that the effect of menstruation was not considered. Since bone metabolism is affected by sex hormones, it is desirable to examine the effect of menstruation in future research.

Based on the findings above and in light of peak height velocity age, we believe that it is important to practice a lifestyle that will increase sufficient height and muscle mass by the third year of junior high school for males and by the fifth grade for females to increase peak bone mass. This requires a regular lifestyle, balanced nutritional intake, and appropriate exercise. We believe that such a lifestyle will lead to the control of childhood obesity, which is one of the factors leading to the need for nursing care. Moreover, developing desirable lifestyle habits in childhood may also encourage people to be healthy. Raising awareness of health promotion in childhood and practicing desirable lifestyle habits may also lead to the suppression of metabolic syndromes, frailty, and locomotive syndromes, which have been identified as a measure for preventing long-term care. To extend healthy life expectancy, it is important to work on these issues from early adolescence.

## Conclusions

Adolescents aged 10/11 years to 14/15 years in the Iwaki area of the Aomori Prefecture were evaluated and followed up for four years. Bone metabolism marker values among participants aged 14/15 years were significantly higher in boys than in girls. At 10/11 years of age for girls, a positive correlation was found between body size/grip strength and OSI. At 14/15 years of age for boys, all body size factors/grip strength were positively correlated with OSI. Change in body muscle ratio was positively correlated with the change in OSI among for both sexes. The height, body muscle ratio and grip strength at 10/11 years of age were significantly positively associated with OSI and significantly negatively associated with bone metabolism markers at 14/15 years of age for both sexes. In conclusion, adequate physique building after 10/11 years of age for boys and before 10/11 years of age for girls may be effective in increasing PBM.

## Data Availability

The datasets used and/or analyzed during the current study available from the corresponding author, S.N., upon reasonable request.
